# Integrating Plant Science and Crop Modeling: Assessment of the Impact of Climate Change on Soybean and Maize Production

**DOI:** 10.1093/pcp/pcx141

**Published:** 2017-09-15

**Authors:** N�ndor Fodor, Andrew Challinor, Ioannis Droutsas, Julian Ramirez-Villegas, Florian Zabel, Ann-Kristin Koehler, Christine H Foyer

**Affiliations:** 1 Centre for Plant Sciences, School of Biology, Faculty of Biological Sciences, University of Leeds, LS2 9JT Leeds, UK; 2 Centre for Agricultural Research, Hungarian Academy of Sciences, 2462 Martonv�s�r Brunszvik u. 2., Hungary; 3 Institute for Climate and Atmospheric Science, School of Earth and Environment, University of Leeds, LS2 9JT Leeds, UK; 4 International Center for Tropical Agriculture (CIAT), km 17 recta Cali-Palmira, Cali, Colombia; 5 CGIAR Research Program on Climate Change, Agriculture and Food Security (CCAFS), c/o CIAT, km 17 recta Cali-Palmira, Cali, Colombia; 6 Ludwig-Maximilians-Universit�t M�nchen, Luisenstrasse 37, 80333 Munich, Germany

**Keywords:** high CO_2,_ photosynthesis, crop production, land use, climate change modeling

## Abstract

Increasing global CO_2_ emissions have profound consequences for plant biology, not least because of direct influences on carbon gain. However, much remains uncertain regarding how our major crops will respond to a future high CO_2_ world. Crop model inter-comparison studies have identified large uncertainties and biases associated with climate change. The need to quantify uncertainty has drawn the fields of plant molecular physiology, crop breeding and biology, and climate change modeling closer together. Comparing data from different models that have been used to assess the potential climate change impacts on soybean and maize production, future yield losses have been predicted for both major crops. When CO_2_ fertilization effects are taken into account significant yield gains are predicted for soybean, together with a shift in global production from the Southern to the Northern hemisphere. Maize production is also forecast to shift northwards. However, unless plant breeders are able to produce new hybrids with improved traits, the forecasted yield losses for maize will only be mitigated by agro-management adaptations. In addition, the increasing demands of a growing world population will require larger areas of marginal land to be used for maize and soybean production. We summarize the outputs of crop models, together with mitigation options for decreasing the negative impacts of climate on the global maize and soybean production, providing an overview of projected land-use change as a major determining factor for future global crop production.

## Introduction

Atmospheric CO_2_ concentrations [CO_2_] have risen from about 280 μLL^−^^1^ in pre-industrial times to 400 μLL^−^^1^ at present (IPCC 2013). The increasing concentration rate has accelerated in recent years to the extent that [CO_2_] may reach between 530 and 970 μLL^−^^1^ by the end of the 21st century, leading to significant global warming (IPCC 2013). Higher temperatures and high [CO_2_] can be both beneficial and detrimental to plants, leading to changes in the global agricultural landscape. Average global temperatures have increased by 0.76�C over the last 150 years and are likely to increase by at least another 1.7�C by the end of this century. It is generally assumed that most plants are adapted to atmospheric [CO_2_] below 300 **μ**LL^−^^1^ and that evolutionary adaptation may not keep pace with ongoing rapid atmospheric CO_2_ increases (Ort et al. 2015).

Since high [CO_2_] will favor photosynthetic carbon assimilation and depress photorespiration in plants with the C3 pathway of photosynthesis, it is generally assumed that C3 plants will benefit from increased carbon gain that will translate into increased biomass and yield. Many aspects of plant metabolism, molecular physiology, structure and development are modified by growth under high atmospheric [CO_2_], not least because the assimilation of carbon is tightly linked to primary nitrogen assimilation (Terashima et al. 2015 and articles cited therein). Moreover, increased [CO_2_] reduces the density of stomata and also decreases the aperture of the stomatal pores resulting in decreased evapotranspiration (Mansfield et al. 1990, Vavasseur and Raghavendra 2005, Kim et al. 2010). Stomatal development is also controlled by both [CO_2_] and the phytohormone abscisic acid (ABA; Woodward 1987, Woodward and Kelly 1995, Tanaka et al. 2013). Several components have been identified in the signaling pathway that reduces stomatal apertures in response to elevated [CO_2_] including β-carbonic anhydrases (Hu et al. 2010), the HT1 protein kinase, the RHC1 MATE transporter and the NtMPK4 protein kinase (Hashimoto et al. 2006, Marten et al. 2008, Tian et al. 2015). The generation of reactive oxygen species (ROS) is involved in both high [CO_2_]-induced decreases in stomatal density, requiring the presence of ABA, PYR/RCAR and ABA receptors (Chater et al. 2015). Despite extensive research efforts over the last 50 years, the complex interplay between metabolic and environmental signals that determine the plant response to high CO_2_ is far from resolved, particularly at the whole plant level. Much of our current understanding of the responses of crop growth to high atmospheric [CO_2_] has come from either studies in free air CO_2_ enrichment (FACE) sites or chamber (closed or open-top) experiments. Unfortunately, such studies have not always yielded consistent results. CO_2_ enrichment does not necessarily enhance plant growth or yield and differences in the responses of these traits have been reported even within the same species (Ainsworth and Long 2005, Luo et al. 2006, Leakey et al. 2009a b, Hasegawa et al. 2013, Bishop et al. 2015). Nevertheless, these studies provide the essential foundation data underpinning crop models, predicting future changes in crop production and their implications for food security.

Crop models have a central role in informing agro-industry and policymakers about the risks and potential of adaptation strategies to counter climate change, as well as directing plant scientists and breeders towards the required traits in improved varieties and cropping systems’ management practices to mitigate global climate change impacts. Crop model inter-comparison studies have identified large uncertainties and biases (e.g. Asseng et al. 2013, 2014, Bassu et al. 2014), and unfortunately they do not often incorporate current knowledge of plant responses to growth under high atmospheric [CO_2_] (Durand et al. 2017). This review summarizes current crop models and the complexity of analysis, within the context of our current knowledge on the impacts of a high [CO_2_] on the C3 crop plant soybean (*Glycine max*), and the C4 crop maize (*Zea mays*), which has an internal CO_2_ concentrating mechanism. Maize and soybean are used to produce a wide range of food and non-food products including pharmaceuticals and biofuels, as well as important sources of livestock feed. Here, we consider the projected impacts of a future high CO_2_ world on the global production of maize and soybean. Currently, maize is the most important grain crop and soybean the fourth most important in terms of global production. Since 1960, soybean and maize grain yields increased 7.6 and 2.6 times, respectively. Together, the USA, Brazil and Asia (mainly China and India) account for respectively 92% and 84% of the world soybean and maize production. However, while the land area on which grain legumes, such as soybean, are grown has gradually increased over the past 50 years, this is still only a quarter of that planted to cereals, such as maize (Foyer et al. 2016). In addition, while increases in cereal production over this period have been predominantly due to increases in yield, driven by the introduction of new varieties and improvements in agronomic practices, increases in grain legume production are due to both increases in land area planted and grain yield (Foyer et al. 2016). For soybean in particular, grain yields have increased in proportion to the land area planted. Moreover, year-on-year increases in soybean yields are slowing while area planted is increasing, suggesting that more marginal land is being planted.

In this review, we will provide a brief overview of our current understanding of the molecular, metabolic and physiological responses of plants to increasing atmospheric [CO_2_] and briefly summarize the history and types of crop models that are currently available. We then specifically address the question of how increasing atmospheric [CO_2_] will alter global soybean and maize production patterns. Using 118 peer-reviewed publications (31 for soybean and 87 for maize), we review the main issues that should be taken into account when modeling these two important crops, namely model inputs, the roles of [CO_2_] adaptation, mitigation, and modeling uncertainties. Finally, we discuss projected land-use change as a major determining factor for future global crop production.

### The plasticity of plant responses to high CO_2_

There is now an extensive literature on the responses of plant biology to growth under high [CO_2_] conditions, with reviews ranging from the control of photosynthetic electron transport and re-programming of photosynthetic gene expression that accompanies the suppression of photorespiration (Foyer et al. 2012) to effects on abiotic stress tolerance (AbdElgawad et al. 2016). It is not our intention therefore to describe the complex and many-faceted responses of plants to CO_2_ enrichment but rather to highlight a few of the salient points that form the basis for current assumptions made in crop models.

Current atmospheres have a CO_2_:O_2_ ratio of 0.00194 but this may increase to values as high as 0.0047 by the end of this century (IPCC 2013), because CO_2_ is currently increasing at an annual rate average of 2.1 **μ**LL^-1^ (Dlugokencky and Tans 2017). This will benefit plants such as soybean that rely on C3 photosynthesis. High atmospheric [CO_2_] in FACE experiments resulted in increased soybean photosynthesis rates of up to 46 % (Leakey et al. 2009a). This enhancement is possible because the current atmospheric [CO_2_] of 400 **μ**LL^-1^ is insufficient to saturate the enzyme responsible for photosynthetic carbon assimilation, ribulose-1,5-bisphosphate carboxylase-oxygenase (Rubisco; Farazdaghi 2011). Gaseous CO_2_ is much more soluble in water than O_2_, and thus the local CO_2_:O_2_ ratio in the chloroplast environment is currently about 0.026 at 25�C. Rubisco has about a 100-fold greater affinity for CO_2_ than O_2_ in higher plants, dictating that this enzyme catalyzes between two and three cycles of carboxylation for every cycle of oxidation. In this way, carbon is partitioned between the assimilatory C3 cycle and the photo-respiratory pathways. Hence, higher CO_2_:O_2_ ratios will competitively inhibit the oxygenase activity of Rubisco and C3 carbon fixation will be favored over photorespiration. However, the potential benefits offered by increased carbon gain are often not fully realized because of insufficient sink capacity when C3 plants are grown at elevated [CO_2_] (Paul and Foyer 2001, Bernacchi et al. 2005). This results in carbohydrate accumulation in source leaves, a signal that causes repression of genes encoding photosynthetic proteins leading to a down-regulation of photosynthesis and a decrease in leaf nitrogen (N) content (Leakey et al. 2009a). Limitations in soil nitrate availability can also lead to down-regulation of photosynthesis in plants grown at elevated [CO_2_]. The ‘progressive N limitation’ hypothesis suggests that under CO_2_ enrichment, plant N uptake from soils fails to keep pace with photosynthesis and shoot carbohydrate accumulation (Foyer et al. 2009). Growth at elevated [CO_2_] can also significantly reduce leaf litter N availability, and lead to poor soil quality because of suppressed decomposition and increased microbial immobilization (Cha et al. 2017). It has also been argued that photorespiration plays an important role in providing the reductant required to drive the assimilation of nitrate into ammonium (Rachmilevitch et al. 2004). Hence, that increasing [CO_2_] will favor C3 plants, particularly in environments where NH_4_+ is available as a nitrogen source. The increase in carbon gain achieved by C3 plants under CO_2_ enrichment may also serve ameliorate problems associated with ammonium toxicity (Li et al. 2014).

The effects of increasing [CO_2_] on plant architecture and partitioning of biomass between roots and shoots remains uncertain. Much depends on the C/N balance in roots and shoots. N-availability signals in the shoot influence the root system. The shoot promotes root growth in proportion to total N-demand. Plant architecture responses to increasing [CO_2_] are likely to involve complex pathways of root-to-shoot and shoot-to-root signaling. Signaling molecules include the small C-terminally encoded peptide (CEP) family peptides, which control root system architecture (Mohd-Radzman et al. 2015) and the CLAVATA signaling pathway, which controls root development and involves small signaling peptides of the clavata3/embryo surrounding region (CLE) family that are important in the regulation of stem cell division and differentiation in an N-responsive manner (Araya et al. 2016). In N-deprived roots CEP peptides are produced and transported to the shoot, where they induce of expression of ‘CEP-downstream’ peptides that are transported back to the root to increase the expression of N-uptake transporters. There is a paucity of literature to date concerning how high [CO_2_] influences whole plant signaling.

One particularly important result of the growth of C3 plants under elevated CO_2_ is the priming of pathogen defenses (Mhamdi and Noctor 2016). Multiple pathogen defense pathways are activated when C3 plants are grown with atmospheric CO_2_ enrichment, leading to increased resistance to bacterial and fungal pathogens. This high [CO_2_]-dependent priming of pathogen defenses is linked to metabolic adjustments involving redox signaling (Mhamdi and Noctor 2016). While growth under elevated [CO_2_] may enhance the resistance/resilience of C3 plants to pests and pathogens, a FACE study showed no effects on aphid performance (Mondor et al. 2005).

C4 plants such as maize are able to concentrate CO_2_ in the Rubisco-containing photosynthetic cells of the bundle sheath. The CO_2_-concentrating mechanism of C4 photosynthesis facilitates high rates of carbon assimilation to occur even when stomata are partially or fully closed, because the C4 pathway delivers a high CO_2_ concentration in the vicinity of rubisco limiting the oxygenation reaction and flux through the photo-respiratory pathway. Hence the C4 pathway of photosynthesis provides a competitive advantage under growth conditions that promote carbon loss through photorespiration, such as high temperatures or decreased water availability (Lopes and Foyer 2011). The transpiration rates and water status of maize leaves, particularly the older leaf ranks, are changed under conditions of atmospheric CO_2_ enrichment even when plants are maintained under well-watered conditions (Prins et al. 2010). Under well-watered conditions, elevated CO_2_ has little effect on the photosynthesis or growth of C4 plants in controlled environment (Soares et al. 2007, Prins et al. 2010) or in the FACE studies (Leakey et al. 2009a, b, Manderscheid et al. 2014). Moreover, the negative impact of drought on yield is attenuated at high CO_2_ because of stomatal closure (Lopes et al. 2011, Manderscheid et al. 2014). Such observations indicate that maize should perform better under drought stress conditions when plants are grown at high [CO_2_]. While higher temperatures should favor C4 plants over C3 plants (Long and Ort 2010), a negative response of global yields has been projected for maize as well as wheat and barley as a result of increased temperatures (Tatsumi et al. 2011, Asseng et al. 2014). Elevated temperatures have been reported to exert a negative influence on a range of plant processes such as photosynthesis through decreased activation of Rubisco, stomatal closure, flower development, pollen viability and hence fertility, and fruit ripening but in many cases the precise mechanisms remain to be characterized.

### The rise of crop modeling

Crop models are designed to calculate crop yield (and other important parameters of the soil-plant system) as a function of weather and soil conditions, plant-specific characteristics as well as a choice of agricultural management practices (see Table 1 for definitions for key terms used hereafter). Models of cropping systems were first conceived in the 1960s (Jones et al. 2017). Although it is fundamentally a curiosity-driven activity, the development of crop models received major boosts from various economic, technological and political events. During the Cold War, fueled by the unexpected large volume purchase of wheat by the Soviet Union in 1972, another type of curiosity played an important role in the development of key components of the DSSAT model suite (Jones et al. 2003) enabling the USA to predict the yield of major crops produced and traded worldwide, especially in the COMECON (Council for Mutual Economic Assistance) countries (Ritchie 2000). The current version of DSSAT (Decision Support System for Agrotechnology Transfer) software application comprises crop simulation models for various cereals, grain legumes and root crops. Outside simulating plant growth, development and yield formation the model is calculating the soil heat, water and nitrogen balance as a function of the soil-plant-atmosphere dynamics and agro-management options. Governmental funds helped experts from different disciplines to develop crop models with new capabilities: EPIC (Williams et al. 1989) with a soil erosion module, APSIM (Keating et al. 2003) able to simulate large number of different crops including trees and weeds. The Environmental Policy Integrated Climate (EPIC) model is a cropping systems model that was developed to estimate soil productivity as affected by erosion. Today, EPIC simulates approximately eighty crops with one generic crop growth model using unique parameter values for each crop. It predicts effects of management decisions on soil, water, nutrient and pesticide movements, and their combined impact on soil loss, water quality, and crop yields. The Agricultural Production Systems Simulator (APSIM) software is a modular modeling framework developed to simulate biophysical processes in agricultural systems, particularly as it relates to the economic and ecological outcomes of management practices with regards to climate risks. APSIM is structured around plant, soil and management modules comprising diverse range of crops, pastures and trees, soil processes including water balance, N and P transformations, soil pH, erosion and a full range of management controls. The release of the first personal computers in the early 1980s revolutionized not only the use and development of crop models but it led to many innovations in other fields (computer graphics, statistical analysis, GIS, etc.) that have contributed to the modeling of agricultural systems (Jones et al. 2017).

**Table 1:** Key definitions

Term	Definition
Greenhouse gases (GHGs)	These are gases (e.g. water vapor, carbon dioxide, methane) in the atmosphere that absorb and emit radiation warming Earth’s surface to a temperature above what it would be without the atmosphere.
Climate change adaptation	In the context of climate change adaptation means taking appropriate actions (e.g. move the planting dates earlier or introducing drought tolerant varieties) to prevent or minimize the damage the adverse effects of climate change can cause, or taking advantage of opportunities that may arise (e.g. expanding cropping areas of certain crops).
Climate change mitigation	In the context of climate change mitigation refers to efforts to reduce or prevent emission of greenhouse gases. Mitigation can mean using new technologies and renewable energies, making older equipment more energy efficient, or changing management practices (e.g. minimize soil cultivation) or consumer behavior.
Representative concentration pathways (RCPs)	These are four greenhouse gas concentration trajectories adopted by the Intergovernmental Panel on Climate Change (IPCC) in 2014. They describe four plausible climate futures, all of which are considered possible depending on how much greenhouse gases are emitted in the years to come. RCP2.6, RCP4.5, RCP6, and RCP8.5, are named after a possible range of radiative forcing values (the difference between the incoming radiation absorbed by the Earth and the energy radiated back to space) in the year 2100 relative to pre-industrial values (+2.6, +4.5, +6.0, and +8.5 W/m2, respectively)
Computable general equilibrium (CGE)	These models are a class of economic models that use actual economic data to estimate how an economy might react to changes in policy, technology or other external factors.
Scopus	This is the world’s largest abstract and citation database of peer-reviewed research literature with over 22,000 titles from more than 5,000 international publishers.
Fuzzy logic	This is a form of multi-value logic in which the truth values of variables may be any real number between 0 and 1. It is employed to handle the concept of partial truth, where the truth value may range between completely true and completely false.

Crop modeling has been used for various applications over the past few decades. Field-scale applications for decision support have a long history (Hoogenboom et al. 1994) that in turn enabled work with seasonal weather forecasting (Hansen 2005), frameworks to link crop and climate models (Challinor et al. 2003), or integrated assessments within watersheds or across multiple sectors (Warszawski et al. 2014, Wriedt et al. 2009). Crop models have been used to develop adaptation options (Webber et al. 2014, Challinor 2009) and there is now recognition of the need for combined assessments of adaptation and mitigation, in support of achieving emissions targets (Jarvis et al. 2011, Shirsath et al. 2017). The need to quantify uncertainty (Challinor et al. 2013) and to improve models has led to an increasing number of international collaborations across modeling groups (Rosenzweig et al. 2013), as well as to linking crop models to climate model ensembles (Ramirez-Villegas et al. 2013). Recognition of the importance of vulnerability and agricultural management in determining impacts and adaptation options has led to work across the natural-social science interface (Simelton et al. 2012). For a detailed history of crop models see the comprehensive work of Jones et al. (2017).

### Major types of crop models

Approaches used to assess the impacts of climate change on agriculture include four major types.

1. Climate or more generally, environmental index-based methods (Olesen et al. 2011) utilize a multidimensional scoring system of production determining factors to provide a quasi-quantitative assessment of the vulnerability of the investigated agricultural system or area.
2. Statistical models express the relationship between yield or yield components and weather parameters in a form of regression equations (Lobell and Burke 2010) or other type of more ‘black-box’ models (Delerce et al. 2016) which are calibrated by using corresponding observed yield and weather data varying in time or space or in both domains.
3. Niche-based models describe the geographical distribution of a crop species using either a set of explicit fuzzy-logic equations that specify how environmental suitability (a continuous variable in the range 0–1) varies across an environmental gradient (Zabel et al. 2014) or a statistical model fitted with presences and absences of the crop in question (Estes et al. 2013).
4. Process-based models (Rosenzweig et al. 2014, Ewert et al. 2015, M�ller et al. 2017) are the mathematical (and nowadays usually computer-based) representation of the most important processes of the soil-plant system consisting of a set of ordinary or partial differential equations and empirical equations organized into procedures or modules where the outputs of one procedure can serve as input to other procedures and the model as a whole is able to describe the temporal pattern of the key system parameters.

That is why these models are also called crop simulation models. Each type of model has advantages and disadvantages as well as limitations. However, all are useful tools when considering the potential impact of climate change. Researchers select the model that best suits the application. From the point of view of the present question, the major limitations of the first three approaches are that they cannot capture future climate-soil-crop relationships, adaptation through crop management and carbon dioxide fertilization effect, though there are techniques to estimate the latter in statistical methods (McGrath and Lobell 2011). Probably this is the main reason why process-based crop models are the most commonly used tools for climate impact assessments (White et al. 2011).

### State of the art of crop modeling

The capabilities of crop models depend in large part on the observed data used for developing and testing the model, and on modeling the crop at a degree of complexity that is appropriate to the aims of the study (Sinclair and Seligman 2000). The results of any one particular study are highly dependent upon input data quality and adequate quantification of uncertainty, though synthesis across many studies helps achieving consensus (Challinor et al. 2014b). Crop model ensembles should represent the underlying distribution of probabilities, which is not straightforward (Wallach et al. 2016). Attention should be paid to bias correction of climate data where necessary (Hawkins et al. 2013). The assumptions underlying the results of the study should be explicit, for example using a common uncertainty reporting format (Wesselink et al. 2015). For adaptation, there are number of issues that need attention when formulating a study (see Lobell 2014).

Whilst the spread of results produced by crop models has increased over time, robust conclusions can still result from analysis of outputs (Challinor et al. 2014b). Crop models are increasingly used for global assessments (Rosenzweig et al. 2014). There are currently two large modeling initiatives, AgMIP (agmip.org) and Modeling European Agriculture for Climate Change (MACSUR: macsur.eu). These networking hubs coordinate and support crop model development, together with crop model based studies and impact assessments, providing information for producers, policy-makers and the public in the area of integrated climate change risk assessment for global agriculture and food security. The projections described for maize and soybean below are results of the collaboration of several groups from the AgMIP and MACSUR modeling initiatives.

Understanding the influence of land use on crop production is an important challenge for such studies (Challinor et al. 2015). Effective use of crop models within integrated assessment models is another important challenge (Ewert et al. 2015). Coupling crop models with computable general equilibrium (CGE; Table 1) models to bring supply and demand of agricultural commodities together under the consideration of global trade is another step forward in the evolvement of crop models that allows further investigations, e.g. on the allocation of cropland and land use change (Mauser et al. 2015). These challenges for the use of crop models do nothing to detract from the need for continued model improvement and representation of processes (Hollaway et al. 2012, Challinor et al. 2014a), particularly where experimental limitations occur (Reich and Hobbie 2013).

### Projections for the future of C3 and C4 crops, focusing on soybean and maize

Crop models have been widely used to estimate the potential impacts of climate change on future agricultural productivity. The protocols of the assessments vary to such an extent that they impose serious limitations to cross-study syntheses and increase the potential for bias in projected impacts (White et al. 2011). Despite this fact, the available results allow us to draw some robust conclusions that are outlined below. With the help of the SCOPUS database, we reviewed 118 peer-reviewed publications (31 for soybean and 87 for maize) that used crop models to investigate the impact of climate change on the production of maize and soybean worldwide in the second half of the 21st century. These modeling studies covered all the most important production areas in America, Asia, Europe and Africa. Using these studies, we summarized the key findings on model inputs, consideration of [CO_2_] response, adaptation and mitigation for both crops.

### Models and key model inputs in the soybean studies

Fifteen different models were used to assess the potential climate change impacts on soybean. However, only two models were used in more than two studies. CROPGRO and EPIC model results were reported in 15 and 4 papers, respectively. Seventeen studies investigated more than one location (from two to 100) within the study area (point-based studies) and 11 studies used the gridded modeling approach covering the total investigated area with a specific spatial resolution. No studies used gridded and point-based estimates jointly. Regarding uncertainty quantification, only two papers used more than one crop model, though this technique helps avoiding model-related biases in the climate change impact projections. Conversely, with the exception of two studies, all used several (two to 72) future climate projections to assess (or show) the uncertainty arising from different climate model- and/or climate change scenario-related issues. The projected temperature rise used in the climate projections (compared to the baseline) varied between 0.9 and 9�C, but the majority of the studies examined the effect of a 2–4�C temperature rise. These temperature changes were associated with an increase in the atmospheric [CO_2_] from 450–700 **μ**LL^−^^1^, although the majority of the papers postulated a [CO_2_] of 550–650 **μ**LL^−^^1^ for the future.

### The effect of high [CO_2_] and adaptation options on future soybean production

Of the literature used in this analysis, six studies failed to consider the direct effect of high [CO_2_] on soybeans. All studies projected yield losses for soybean, which might be mitigated by agricultural management adaptations such as changing the planting date (do Rio et al. 2016), change of cultivars (Battisti et al. 2017) or introducing double-cropping systems (Lant et al. 2016). The global study of Tatsumi et al. (2011) projected yield decrease for all the major soybean producing areas. However, this study applied several significant simplifications such as use of monthly step climatic data, ignoring CO_2_ fertilization effects and the water-holding capacity of soils. Twenty-seven studies that took into account CO_2_ fertilization effects projected significant yield gains. Of these, only one global assessment that took into account the added carbon gain arising from future high atmospheric [CO_2_], projected moderate (5–15%) yield losses and this was only for regions in US and Latin-America (Deryng et al. 2014). The same study did not investigate the potential of management adaptation options. In relation to adaptation, in fact, we find that some 16 out of the 37 studies investigated adaptation options. These studies suggest that management adaptation options can have a significant effect in counterbalancing the negative effects of climate change (Tubiello et al. 2000, Challinor et al. 2014b). According to some simulations, some crop management options (e.g. winter rye cover) have no effect on future yields but they have the potential to reduce soil erosion and nitrous oxide emissions significantly (Basche et al. 2016).

### The role of climate change mitigation policies in future soybean production

Representative concentration pathways (RCPs) are four greenhouse gas concentration trajectories (IPCC 2013), all of which are plausible depending on how much greenhouse gases are emitted in the years to come. The four RCPs, RCP2.6, RCP4.5, RCP6, and RCP8.5, are named after the prospective radiative forcing values in the year 2100 relative to pre-industrial values (+2.6, +4.5, +6.0, and +8.5 Wm^−^^2^ respectively). The ultimate aim of climate change mitigation policies is to reduce emissions consistent with specific targets, thus helping to avoid high-end emissions scenarios such as RCP8.5. The Paris Agreement (2015), for example, aims at maintaining global average temperature well below 2�C above pre-industrial levels; this has been reported to significantly reduce the risks and impacts of climate change (Schleussner et al. 2016). This aim could be achieved in many ways including the use of low-carbon technologies, renewable energy sources, transportation optimization, as well as promoting individual-lifestyle changes (cycling instead of driving, alternative diets, etc.). In the agricultural sector, climate change mitigation policies may be implemented via promoting reforestation, low input soil management, resource efficient farm management, more sustainable fertilizer subsidy provision, and improving knowledge and transfer mechanisms all aiming at increasing carbon sequestration and/or decreasing greenhouse gas emissions. Climate mitigation policies play an important role in ensuring that new technologies are implemented and mitigation targets are met. They are hence central to avoiding future global yield losses.

Across the soybean studies reviewed here, mitigation policies are typically addressed by modeling crop yields for different RCPs. Comparison between different RCPs allows determination of the likely benefits of climate change mitigation. For example, the yield reduction reported by Deryng et al. (2014) was the result of using the most extreme RCP8.5 based climate projections which is in fine agreement with the findings of Bhattarai et al. (2017) who, on the other hand, used not only RCP8.5 but RCP2.6 and RCP4.5 based projections resulting in marginal yield losses (−2%) for RCP8.5 and yield gains (11 and 13%) for RCP2.6 and RCP4.5, respectively. The studies reviewed here thus strongly suggest that successful climate change mitigation policies that secure the future [CO_2_] pathway below RCP4.5, will allow future resolution of soybean production problems.

Another important aspect of future crop production is the extent to which areas where crops are grown may shift as conditions change. Some studies have shown that there is a large northerly and southerly shift in land that is suitable for soybean production (Lant et al. 2016). This shift incorporates significant areas of the Northern hemisphere reaching as far as Ireland (Holden and Brereton 2003). Soybeans are already grown in Canada and varieties are already being trialed for production in the UK. Thus, due to the projected future yield and sowing area gains an expansion of soya production could be expected worldwide, although as with projected yield changes, these shifts in production areas could change depending upon the emissions pathway.

### Models and key model inputs in the maize studies

Twenty-one different models were used for assessing the potential climate change impacts on maize. The two most frequently used models were the CERES (member of the DSSAT model suite) and EPIC that were used in 45 and eight studies, respectively. About a third (23) of the assessments were based on data of only one particular site of the study area and/or applied only one climate projection for the future. The projected temperature rise and the associated atmospheric carbon dioxide increase of the climate projections of the maize studies were similar to those of the soybean studies. Regarding crop model uncertainty, twenty-two studies used the gridded modeling approach and five papers used more than one crop model for the impact assessments. The most comprehensive of these was the study of Bassu et al. (2014), which evaluated 23 maize simulation models for four locations representing a wide range of maize production conditions in the world. They found that only an ensemble of models (a minimum of about eight to 10 needed) was able to simulate absolute yields accurately and that there was a large uncertainty in the yield response to [CO_2_] among models. The uncertainty envelope is mainly due to inconsistency in the way models simulate assimilation, as well as in whether or not models simulate enhanced [CO_2_] effects on transpiration.

### Model and scale related uncertainty in the maize studies

In a global study, Blanc and Sultan (2015) showed that the projected changes for maize production were highly model-dependent, ranging from a 15% decrease to a 20% increase in yield in the Corn Belt. However, large scale investigations may contradict local (country scale) studies even if the same model was used. For example, Supit et al. (2012) projected a yield increase for Turkey as a result of climate change while Sen et al. (2012) predicted that yields will decrease in this region. One reason for this kind of discrepancy could be the lack of use or quality in the soil data used for yield projections (Tatsumi et al. 2011). The impact of climate change on specific regions could vary significantly because of differences in soil characteristics (Chipanshi et al. 2003). Surprisingly, no local model-based impact studies were found for France, Indonesia, Ukraine or South-Africa, although these countries are among the top 10 global maize producers.

### Prospects for future maize production

While a number of studies have predicted increases in maize yields in the major corn-producing areas of the world such as the USA (Tubiello et al. 2002), China (Guo et al. 2010) and Argentina (Travasso et al. 2009), most studies have projected global decreases in maize yields (Schlenker and Roberts 2009, Byjesh et al. 2010, Supit et al. 2012, Deryng et al. 2014, Lin et al. 2015), even in studies that took the beneficial effect of CO_2_ fertilisation into account. Many studies accounted the predicted yield reduction by one or more of the three main reasons: (i) Increasing frequency and severity of drought; (ii) Increasing risk of heat waves around flowering; (iii) Shortening of the vegetation period. However, it may also be the case that current models fail to account for the water-saving mechanisms afforded by C4 metabolism and physiology appropriately. Higher water use efficiencies would be expected in maize under high [CO_2_]. Thus, models failing to take this feature into account might underestimate biomass and yield gains under high [CO_2_]. Durand et al. (2017) assessed the accuracy of maize crop models in simulating the interactions of changes at high atmospheric [CO_2_]. Under well-watered conditions the models were able to reproduce the absence of yield response to elevated [CO_2_]. However, under water deficit conditions the models failed to capture the extent of the [CO_2_] response that was observed in the field (Deryng et al. 2016).

Regional gridded modeling studies are particularly important in maize yield projections because they are able to distinguish between sub-regions that may be positively or negatively affected by climate change. The currently high yielding sub-regions of China may face yield decreases while the current low yielding sub-regions may expect yield increase (Xiong et al. 2007). Current high yielding sub-regions are near-optimum zones providing very favorable conditions for maize production. Almost any environmental change in these areas could only be negative as it would distance the system from its near-optimum state. On the other hand, marginal areas (far from the optimum) most likely benefit from the environmental changes, by getting closer to the optimum state of the system. However, yield losses per unit area do not necessarily translate into overall productivity for a given region, because the projected area of cultivated land used for multiple-cropping systems may be significantly increased as a result of climate change (Yang et al. 2015). Moreover, the indirect effects of climate change can become important; for example, the projected increases in insect pests as a result of increased winter survival (Diffenbaugh et al. 2008). Such factors could significantly alter the pest management landscape of North American maize production, leading to substantial economic impacts through increased seed and insecticide costs, as well as decreased yields.

### Roles for adaptation options and climate change mitigation policy in future maize production

Modeling studies do not depict a clear positive or negative picture for future global maize production but they clearly emphasize the need for explicit adaptation actions such as breeding of heat/drought tolerant hybrids. The majority of the studies (13 out of 20) that assessed certain adaptation options concluded that a shift in planting date, together with the use of longer maturing hybrids and alternative soil and nitrogen management practices will be insufficient to counter negative impacts of climate change (Tubiello et al. 2000, Ko et al. 2012, Moradi et al. 2013). Studies also agree that the more extreme the scenario (RCP8.5 or similar scenarios form the earlier IPCC reports) the more severe the yield losses that could be expected. This highlights the necessity and opportunities for joint mitigation-adaptation efforts. A global study suggest that the drastic climate mitigation policy of RCP2.6 could avoid more than 80% of the projected global average yield losses (USA: −20%, Brazil: −50%, Argentina: −40%) that are otherwise projected by the 2080s under RCP8.5 (Deryng et al. 2014).

### Methods used to project land use changes for maize and soybean production

**Fig. 1:**
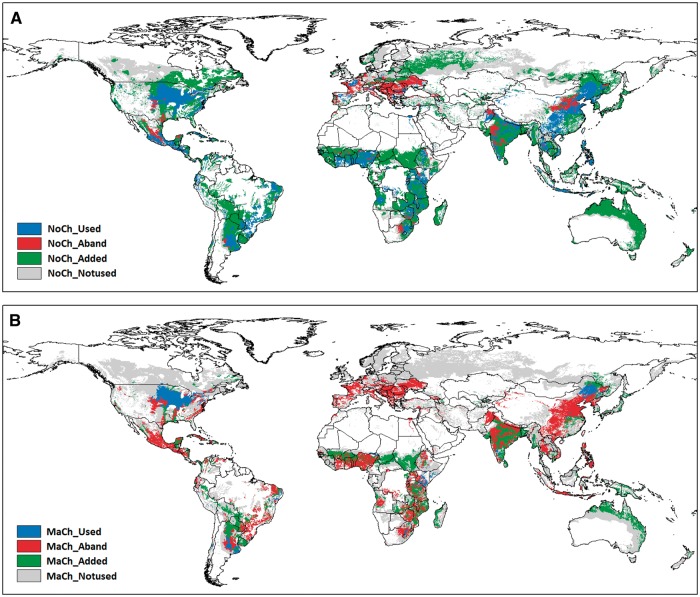
Current maize growing areas (blue), together with predicted abandoned (red) and added (green) maize growing areas by 2100. Gray shade shows the areas that are not used for producing the specific crop. The ‘No change’ scenario (A) is the extrapolation of the current trends with no major GHG emission reductions or no major changes in dietary trends that would result in an increasing need for maize production. The ‘Major change’ scenario (B) will be attained if successful GHG mitigation policies are enforced and significant health-driven changes in diets occur that result in a decreasing need for maize production.

**Fig. 2:**
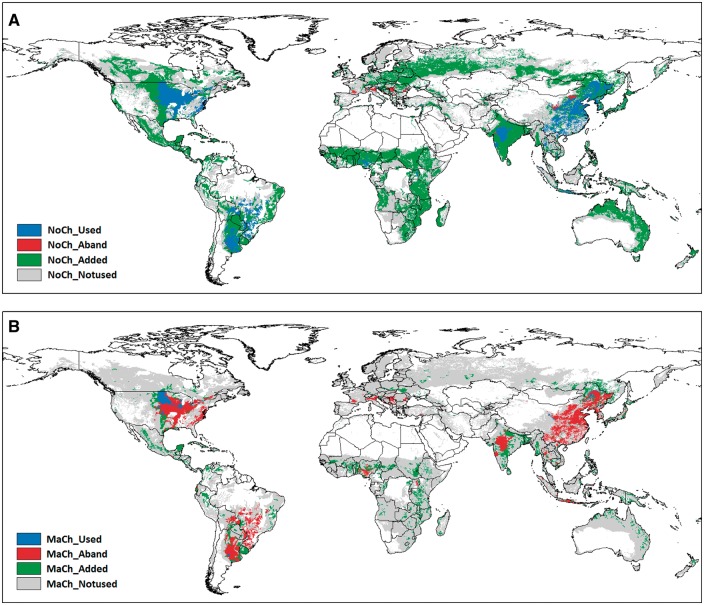
Current soybean growing areas (blue), together with predicted abandoned (red) and added (green) soybean growing areas by 2100. Gray shade shows the areas that are not used for producing the specific crop. The ‘No change’ scenario (A) is the extrapolation of the current trends with no major GHG emission reductions or no major changes in dietary trends that would result in an increasing need for soybean production. The ‘Major change’ scenario (B) will be attained if successful GHG mitigation policies are enforced and significant health-driven changes in diets occur that result in a decreasing need for soybean production.

Coupling land use (Monfreda et al. 2008) and baseline and future land suitability data (Zabel et al. 2014) with future diet (Tilman and Clark 2014) and GHG emission (Smith et al. 2008) scenarios we have projected future of global maize (Fig. 1) and soybean (Fig. 2) production areas. Baseline (1981–2010) and future (2071–2100) land suitability determinations for each grid cell were made using the fuzzy-logic methodology of Zabel et al. (2014) by incorporating data on local daily climate (temperature, precipitation, solar radiation), soil (texture, hydraulic characteristics, pH, organic carbon content, salinity, sodicity) and topography (elevation, slope). We consider 16 economically important staple and energy crops (including maize and soybean) at a spatial resolution of 30 arc seconds. The parameterization of the membership functions that describe each of the crops’ specific natural requirements is taken from Sys et al. (1993). As a result of the fuzzy logic approach, values in a range between 0 and 1 describe the suitability of a crop for each of the prevailing natural conditions at a certain location. The smallest suitability value over all parameters finally determines the suitability of a crop. Daily climate data are taken from the global climate model ECHAM5 (Jungclaus et al. 2006) for SRES A1B climate scenario conditions. Soil data are taken from the Harmonized World Soil Database (FAO et al. 2012), and topography data are retrieved from the Shuttle Radar Topography Mission (Farr et al. 2007).

The ‘No Change’ scenario is the extrapolation of the current trends, i.e. assuming that no major GHG emission reductions will be achieved by the introduction of mitigation policies or enhanced climate-smart agro-technologies. Moreover, the scenario predicts that increases in income and urbanization will drive a global dietary transition that involves increasingly higher consumption of refined sugars, fats, oils and meats (Tilman and Clark 2014). Together, these features will result in increased demands for maize and soybean production. In contrast, the ‘Major Change’ scenario envisages successful and effective GHG mitigation policies, together with the instigation of new GHG emission reducing agricultural practices. This will involve significant health-driven changes in diets and adoption of alternative diets such as Mediterranean, pescetarian or vegetarian diets that are characterized by higher consumption of fruits, vegetables and pulses and a lower meat consumption (Tilman and Clark 2014). The vast majority of soybean (75%) is currently used to feed livestock, with only about 6% used directly as human food. Future decreases in meat consumption will therefore lead to large decreases in soya demand. Global crop production area maps were created using these scenarios with a 10 km (5 arc minute) spatial resolution. According to current land use (LU) given by (Monfreda et al. 2008) each grid cell can have two states: used (harvested area fraction of the crop is at least 1% of the grid cell area) and not used (Table 2).

**Table 2:** Rules of projections of future of crop production areas. LSt, land suitability today (1981–2010); LSf, Land Suitability in the future (2071–2100); PERC33(LSt) and PERC67(LSt), 33rd and 67th percentile of the distribution of the LSt values of the grid cells used for maize/soya production over the global grid. LU denotes land use. Acronyms refer to certain areas with different colors in Figs 1 and 2

Scenario	No change			
LU today	Used	Used	Not used	Not used
LU in the future	Used	Not used	Used	Not used
LU change	Unaltered	Abandoned	Added	Unaltered
Rule	If	If	If	If
	LSf > 0.9 � LSt	LSf ≤ 0.9 � LSt	LSf > PERC33(LSt)	LSf ≤ PERC33(LSt)
Acronym	NoCh_Used	NoCh_Aband	NoCh_Added	NoCh_Notused
**Scenario**	**Major change**			
LU today	Used	Used	Not used	Not used
LU in the future	Used	not used	Used	Not used
LU change	Unaltered	Abandoned	Added	Unaltered
Rule	If	If	If	If
	LSf > 1.1 � LSt	LSf ≤ 1.1 � LSt	LSf > PERC67(LSt)	LSf ≤ PERC67(LSt)
Acronym	MaCh_Used	MaCh_Aband	MaCh_Added	MaCh_Notused

The objective of the projections shown in Fig. 1 and Fig. 2 is to highlight likely changes in land use patterns. The crop production scenarios reported here predict significantly different demands for land use for maize (Fig. 1) and soybean (Fig. 2) production. Both the used and the not-used cells may remain in the same land use category or may be changed in the future providing four options that can be defined by certain rules for both scenarios (Table 2). If land is ‘used’ today according to the definitions used above, we assume that these areas will be unaltered in the future (2071–2100) in the ‘Major Change’ scenario, if the suitability increases by at least 10%. If suitability increases less than 10% or decreases until 2071–2100, we assume that these areas will be abandoned and not be used in the future.

Crucially, areas that are currently not used for maize (Fig. 1B) and soybean (Fig. 2B) production will probably be added if future land suitability is higher than the 67th percentile of today’s global suitability on used areas. Conversely, areas that are currently not used will also not be used in the future if suitability is lower than the 67th percentile. Since demands for soybean and maize production are higher in the ‘No Change’ scenarios than in the ‘Major Change’ scenarios, more areas will be required for the production of these crops. Accordingly, we assume lower thresholds for future land suitability, as well as lower percentiles of suitability on today’s production areas for maize and soybean respectively. Hence, greater areas of marginal land will have to be used for the cultivation maize and soybean in order to fulfill increasing demands.

## Conclusions and Perspectives

Future land use maps were created for maize and soybean using the basic rules outlined in Table 2 (Fig. 1). Major changes in policy, agricultural practice and diet imply that major shifts will occur in the area used for maize and soya production. Our assessment of modeling outputs predicts that large portions of current areas of significant maize and soya production may be abandoned in the future. On the other hand, large new areas will become available in the future (Table 3) in order to meet the increasing demands on maize and soya production, particularly if no significant policy, agro-technological and diet-related changes take place in the future. According to the projections Europe will face major challenges in both production scenarios, especially in case of maize. Aligned to other studies (Ruiz-Ramos and M�nguez 2010, Supit et al. 2012, Fodor et al. 2014, Mihailović et al. 2015) a stern warning sign could be given to the European Union that effective adaptation actions are required to mitigate the harmful impacts of climate change across the continent. At the other end of the spectrum is Africa, where climate change may allow a massive increase in soybean production no matter which production scenario becomes a reality in the future. It is not surprising that soybean is called Africa’s Cinderella crop (Kolapo 2011). The studies that were assessed here predict a more promising future for soybean, particularly in terms of production areas, gained and abandoned (Table 3). These crop models provide essential underpinning information to farmers, agro-industries and policymakers, so that appropriate cropping systems and/or management practices can be put in place to counter global climate change.

**Table 3:** Predicted global gains and abandoned areas of maize and soya production. The ‘No change’ scenario is the extrapolation of the current trends with no major GHG emission reductions or no major changes in dietary trends that would result in an increasing need for maize or soybean production. The ‘Major change’ scenario will be attained if successful GHG mitigation policies are enforced and significant health-driven changes in diets occur that result in a decreasing need for maize or soybean production

Scenario	Transition	Acronym (see Fig. 1)	Maize [km^2^]	Soya [km^2^]
No change	Abandoned	NoCh_Aband	3,364,115	299,005
	Added	NoCh_Added	27,740,977	30,524,853
Major change	Abandoned	MaCh_Aband	13,287,592	6,506,380
	Added	MaCh_Added	10,137,774	6,547,211

Crop models have an important role to play in informing plant scientists and breeders of essential traits that must be developed in future crop varieties. However, there is a wide gulf between plant science and crop modeling such that much of our current knowledge of plant responses to elevated atmospheric [CO_2_] is not taken into account in many current models. Crucially, current models do not incorporate the latest findings about how crops respond to a changing climate. There is therefore an urgent need for a new interface of information exchange between crop modelers and plant scientists highlighting weaknesses and overlooked processes, and to influence how models are built, to include how recent changes in our understanding of [CO_2_]-mediated effects on plants might be formalized and incorporated into models. It is thus timely to renew discussions in order to remove the large uncertainties and biases in some current crop models, as well as informing plant scientists of the essential underpinning traits that will ensure food security over the next 50 years. Current crop varieties are not well suited to future unpredictable weather patterns caused by climate change. Modern breeding programs have selected for dwarf shoot systems, minimizing the production of vegetative tissues. Moreover, elite crop varieties are developed and bred under ideal growth conditions so the selective pressure for plant performance under sub-optimal conditions has largely been removed. This has favored small root systems, a trait that may have inadvertently decreased the resilience of plants to both abiotic and biotic stresses, which are likely to increase as a result of climate change.

Finally, plant physiologists should be aware of areas where collaboration and data generation would greatly assist crop modelers:

1. *Grain quality aspects:* While FACE experiments clearly indicate that CO_2_ enrichment affects grain quality characteristics that are important for consumer nutrition and health, and for industrial processing and marketing (H�gy et al. 2009), CO_2_ enrichment effects on grain quality traits remain poorly characterized in terms of metabolite, proteome and transcript profiles. Some field-scale crop models already include yield quality related outputs, including sugar and acid concentrations (Bindi and Maselli 2001), grain protein (Asseng et al. 2002) and grain protein composition (Martre et al. 2006) protein composition. The yield quality calculation methods that are embedded in the models are often not thoroughly tested, especially not by using data from elevated CO_2_ experiments. While manipulation of some of the enzymes of primary carbon assimilation was found to protect soybean seed yields against the negative effects of elevated temperature on plants grown at high CO_2_ (K�hler et al. 2016), there are no comparable studies in the literature on effects on grain quality.
2. *More accurate vegetation-related to CO_2_ fluxes:* An important aspect of the crop simulation models typically used for climate change impacts assessments is that they harness important, widely validated knowledge on crop responses to biotic and abiotic factors (Boote et al. 2013). Recent progress in crop, ecosystem, and climate modeling has led to integration of these disciplines in support of integrated assessments of agro-ecosystems at the global or regional level (e.g. Osborne et al. 2007, 2015, Wang et al. 2005). In these cases, crop models may provide the underlying information, parameters and mathematical formulations that underpin the vegetation models used. Nevertheless, much work remains to be done in crop simulation models if these are to be fully integrated within vegetation models. Foremost, adequately simulating vegetation within complex agro-ecosystems requires detailed consideration of CO_2_ uptake for gross primary productivity and CO_2_ release through respiration (Cramer et al. 2001). While progress has been made in developing and testing leaf-to-canopy assimilation in some crop models, only a handful of models for the major crops, including maize and soybean, include detailed photosynthesis-respiration routines for both assimilation and CO_2_ fertilization (Bassu et al. 2014, Li et al. 2015). Moreover, respiration costs associated with the maintenance of existing tissue (maintenance respiration) and the production of new tissue (growth respiration) are either highly uncertain or not estimated or reported in crop simulation studies. Furthermore, testing of CO_2_ fluxes or canopy assimilation using eddies of air, although feasible, is rarely if at all conducted for crop simulation models (Hollinger et al. 2005, Paul et al. 1999). Finally, appropriate consideration and validation of CO_2_ fluxes in crop models will also help improving the calculation of water fluxes and evapotranspiration, which is a key source of uncertainty in crop simulation (Liu et al. 2016).
3. *Canopy temperature and evapotranspiration:* The importance of models predicting global warming effects on crop yield to include canopy temperature instead of using air temperature was demonstrated by Julia and Dingkuhn (2013). They found that rice panicle temperature varied between 9.5 below and 2�C above air temperature at 2 m depending on the microclimate and therefore heat stress causing sterility was more likely to occur in warm-humid than hot-arid environments due to humidity effects on transpiration cooling. Even though some crop models calculate canopy from air temperature, which is then used on some but not necessarily all temperature-related processes in the crop model, Webber et al. (2015) found that this did not necessarily improve yield simulations. The study compared nine process-based crop models that used three different approaches of simulating canopy temperature (empirical, energy balance assuming neutral atmospheric stability, and energy balance correcting for the atmospheric stability conditions) in their ability to simulate heat stress in irrigated wheat in a semi-arid environment. Methods assuming neutral atmospheric stability determine the resistance of the surface to transfer water vapor and heat to the air as a function of crop height and wind speed whereas methods correcting for atmospheric stability include canopy temperature in the calculations. They found that for all models the reduction in the root mean square error was larger if canopy temperature was only used for the processes simulating heat stress but that using canopy temperatures for all processes did not necessarily improved yield simulations. Models that performed well in simulating yield under heat stress had varying skill in simulating canopy temperature (the method energy balance assuming neutral atmospheric stability performed worst). Models differ in parameter values which might be able to somewhat alleviate the impact from using air temperature. Unfortunately the models could not be tested with observed canopy temperature as it was not measured continuously throughout the growing season. Webber et al. (2015) concluded that a more systematically understanding of heat stress events and how to model them is needed.
4. *Effects of high ozone concentrations:* Ozone is highly phytotoxic and can cause significant damage to vegetation and crops even at current concentrations in many parts of the world (Wang and Mauzerall 2004, Booker et al. 2009, Mills et al. 2011, Hollaway et al. 2012). Both maize and soybean are sensitive to ozone (McGrath et al. 2015), with predicted global yield losses ranging from 2.5–8% for maize and 9.5–15% for soybean for the year 2030 (Avnery et al. 2011). However, the negative effects of ozone are included only in a few crop models. For example, the WOFOST model accounts for ozone damage to crops by using a flux-based approach in which the ozone flux inside the plant is regulated by the stomatal conductance (Cappelli et al. 2016). The model shows that for wheat there are large yield losses under high ozone exposure (i.e. up to 30% loss for ozone concentration of 60 ppb; Cappelli et al. 2016). While the effects of ozone on plant biology have been extensively studied, the effect of pollution on crop productivity and quality is an important area for future work, particularly as global ozone concentrations are projected to remain at high levels (Fowler et al. 2008). The responses of plants to atmospheric ozone should be assessed in combination with other stresses to address current as well as the future responses under climate change.
5. *Acclimation to elevated CO_2_:* Current knowledge of how plants sense and signal changes in atmospheric [CO_2_] other than effects on photosynthesis, is limited. Moreover, much remains uncertain concerning the mechanisms that define many of the observed plant responses to increased atmospheric [CO_2_] or how these mechanisms will influence biotic and abiotic stress responses under field conditions. In particular, relatively little is known about how high [CO_2_] will influence the soil microbiome or plant interactions with beneficial fungi and bacteria.

## Abbreviations

ABA: abscisic acid
AgMIP: the agricultural model inter-comparison and improvement project
C3: three carbon
C4: four carbon
CEP: C-terminally encoded peptide
CLE: clavata3/embryo surrounding region
FACE: free air CO_2_ enrichment
MATE: multidrug and toxin extrusion
MACSUR: modeling European agriculture for climate change
PYR/RCAR: pyrabactin resistance1 (pyr1)/pyr1-like (pyl)/regulatory components of aba receptors (rcar)
RCH1: resistant to high CO_2_
Rubisco: ribulose-1, 5 carboxylase oxygenase
ROS: reactive oxygen species
